# Monocyte Distribution Width (MDW) as an Early Investigational Marker for the Diagnosis of Sepsis in an Emergency Department of a Tertiary Care Hospital in North India

**DOI:** 10.7759/cureus.30302

**Published:** 2022-10-14

**Authors:** Neeraj Singla, Aditya Jandial, Nalin Sharma, Navneet Sharma, Mandip Bhatia, Ashish Behera

**Affiliations:** 1 Internal Medicine, Postgraduate Institute of Medical Education and Research, Chandigarh, Chandigarh, IND

**Keywords:** total leucocyte count, shock, monocyte distribution width, sepsis, early

## Abstract

Background

Sepsis is an emergency state in response to an infectious process ultimately leading to multiorgan dysfunction and death. There is an urgent need for sepsis detection methods, especially in emergency settings. To fill this gap, monocyte distribution width (MDW) was studied as an early indicator of sepsis.

Aim

To evaluate MDW as an early marker of sepsis.

Material and methods

This was a prospective observational study including critically ill adult patients who presented to the emergency department. MDW was measured using a DxH 900 Hematology Analyser (Beckman Coulter Inc., Miami, FL). Abnormal MDW (>20.0) was considered a predictor of sepsis.

Results

A total of 148 patients were included and categorized according to the Sepsis-2 and Sepsis-3 criteria, as having sepsis (25.6%), sepsis with shock (21.6%), and non-sepsis (52.8%). In patients with sepsis with and without shock, MDW was 28.28 ± 9.20 and 28.02 ± 9.01, respectively, significantly higher than in patients without sepsis (p < 0.001). The diagnostic accuracy value of MDW testing for early sepsis detection was highly significant (0.74, p < 0.000).

Conclusion

MDW can be used as a marker for the early prediction of sepsis.

## Introduction

Sepsis is one of the foremost causes of in-hospital mortality, perturbing the healthcare systems globally [1]. As per the current guidelines, prompt identification of sepsis and initiation of appropriate management in emergency settings is an utmost priority to reduce mortality [2-4]. Prior studies on the efficacy of various biomarkers in expediting sepsis determination have met with limited success [5-7]. Total leucocyte count (TLC) is one of the earliest laboratory tests that is accessible to clinicians in emergency settings [8], and it is regularly used for emergency identification of sepsis, but, unfortunately, elevated TLC is not specific to sepsis. A sepsis-specific biomarker that is routinely available in the emergency department (ED) is urgently needed. It is known that immune cells (neutrophils and monocytes) increase not only in number but also in size during the clinical progression from a localized infection to sepsis and septic shock. Prior studies have shown that the incorporation of parameters reflecting volumetric changes in immune cells improves the diagnostic accuracy of sepsis. MDW reflects the variation in the size of circulating monocytes. It has shown superior performance in differentiating sepsis from other acute illnesses in emergency settings when used alone or in combination with other established clinical parameters like TLC, systemic inflammatory response syndrome (SIRS), and quick sequential organ failure assessment (qSOFA) [9-11].

MDW is a routinely reported component of the automated complete blood count (CBC) performed by hematology analyzers. There is limited literature from India on the utility of MDW as an indicator of sepsis. Overwhelming patient load and scarcity of health resources are among the major challenges that are faced in resource-limited settings. The availability of an inexpensive and readily available biomarker like MDW would help emergency clinicians to identify sepsis and start appropriate treatment promptly.

However, before contemplating the widespread use of MDW in Indian patients, it is prudent to determine its value as a sepsis predictor in a systematic manner. Hence, in this pilot study, we aimed to determine the utility of MDW, alone or in combination with other clinical parameters, in improving the early detection of sepsis in emergency medical services.

## Materials and methods

Acutely ill adult patients who presented to emergency medical services at a tertiary hospital during the study period were prospectively enrolled in this study after the institutional research body granted permission.

Based on clinical data and laboratory parameters at presentation, Using clinical data and laboratory parameters at enrolment, and based on the Sepsis-2 and Sepsis-3 criteria defined using SIRS and qSOFA, respectively [12-14], patients were categorized as having sepsis with shock, sepsis, and non-sepsis.

The occurrence of infection was determined on the basis of investigations and case history obtained within the first 12 hours of presentation to the ED. To characterize sepsis, it was ensured that sepsis criteria were met and that complete blood counts were measured in patients with suspected infection within 12 hours of the emergency presentation. Sepsis with shock was defined with mean arterial pressure (MAP) <65 mmHg, and MDW was calculated as part of CBC by automated hematology analyzer DxH 900 (Beckman Coulter Inc., Miami, FL) within 24 hours of admission. MDW >20.0 was considered abnormal.

For the purpose of the statistical analysis, the primary objective was to determine whether abnormal MDW, either alone or along with TLC, facilitates early sepsis detection by Sepsis-2 criteria and the secondary objective was to determine whether abnormal MDW increases early sepsis detection by Sepsis-3 criteria.

Continuous and categorical baseline characteristics were compared between patient groups as per Sepsis-2 and Sepsis-3 criteria. Continuous, normally distributed variables were compared with the student t-test, and for non-normally distributed variables, the Mann-Whitney U-test was used.

The added value of MDW in relation to other parameters for detecting sepsis was calculated by comparing the areas (AUC) under the ROC curves. The AUC was determined for one predictor variable logistic model using TLC as a predictor of sepsis. Similarly, the AOCs were also determined for two predictor variables logistic model with both TLC and MDW as predictors of sepsis. The AUCs from the two models (TLC vs TLC + MDW) and their confidence intervals (CIs) were compared. On ROC curves, 0.7 is the threshold between poor and fair diagnostic accuracy”.

All performed statistical tests were two‑tailed. Statistical analysis was performed using SPSS 22.0 software (IBM Inc., Armonk, NY).

## Results

This study enrolled 148 patients, including 53 females (35.8%) and 95 males (64.1%), with a median age of 48.73±16.17 years, irrespective of gender. On the basis of Sepsis-2 and 3 criteria, the patients were divided into the categories sepsis (n=38, 25.6%), septic shock (n=32, 21.6%), and non-sepsis (n=78, 52.7%). The sites of infection, in descending order of occurrence, included the abdomen, urinary tract, central nervous system, skin, and soft tissues. Also included were puerperal sepsis and tubercular pleural effusion (Table 1).

**Table 1:** Demographic details and co-morbidities, site of infection, and mortality at 30 days of the patients included in the study

	Non-sepsis	Sepsis	Sepsis with shock	Total
Number of patients (N)	78(52.7%)	38(25.6%)	32(21.6%)	148
Median age (years) (Mean ± SD)	50.72 ± 15.66	40.63 ± 15.67	53.50 ± 14.96	48.73 ± 16.17
Female	26(49.05%)	16(30.18%)	11(20.75%)	53(35.81%)
Male	52(54.7%)	22(23.1%)	21(22.1%)	95(64.18%)
Comorbidities				
Hypertension	25(71.4%)	2(5.7%)	8(22.8%)	35(23.6%)
Diabetes mellitus	13(54.1%)	2(8.3%)	9(37.5%)	24(16.2%)
Chronic kidney disease (CKD)	12(70.5%)	0	5(29.4%)	17(11.4%)
Chronic liver disease (CLD)	16(57.1%)	5(17.8%)	7(25%)	28(18.9%)
Coronary artery disease (CAD)	6(75%)	0	2(25%)	8(5.45%)
Smoking	3(75%)	1(25%)	0	4(2.7%)
Alcoholic	17(68%)	3(12%)	5(20%)	25(16.8%)
Site of infection				
Abdomen		23(62.1%)	14(37.8%)	37(25%)
Urinary tract		4(30.7%)	9(69.3%)	13(8.7%)
Central nervous system		9(90%)	1(10%)	10(6.7%)
Skin and soft tissue			5(100%)	5(3.3%)
Infective endocarditis		0	1(100%)	1(0.67%)
*Hospital stay (days)	8.21 ± 8.12	8.08 ± 7.48	9.33 ± 7.50	8.51 ± 7.72
*30-day mortality, n (%)	11(44%)	4(16%)	10(40%)	25

Values of total leucocyte count (TLC) count and absolute neutrophil count (ANC) were significantly raised in septic patients and septic shock patients compared with non-septic patients (p < 0.001). In patients with sepsis with or without shock, MDW was significantly elevated (p = 0.001) (Table 2).

**Table 2:** Comparison of patients with non-sepsis, sepsis, sepsis with shock on basis of total leucocyte count, absolute neutrophil count, and monocyte distribution width

	Non-sepsis (N = 78)	Sepsis (N = 38)	Sepsis with shock (N = 32)	Total (N = 148)	P value	
Hemoglobin (g/dL)	9.98 ± 2.79	10.33 ± 2.34	9.14 ± 2.53	9.89 ± 2.64		0.156
Total leucocyte count (/µL)	10419.23 ± 5442.3	12339.47 ± 5670.43	16137.50 ± 10674.59	12148.65 ± 7259.32		0.001
Platelet count (/µL)	193179.49 ± 133173.82	238078.95 ± 142987.69	186281.25 ± 139976.32	203216.22 ± 137833.41		0.190
Neutrophils (%)	73.13 ± 11.76	75.26 ± 13.78	76.38 ± 15.64	74.38 ± 13.18		0.451
Lymphocytes (%)	15.84 ± 9.24	15.32 ± 13.55	14.87 ± 13.88	15.50 ± 11.46		0.916
Monocytes (%)	9.07 ± 5.03	7.55 ± 4.03	7.11 ± 3.36	8.26 ± 4.53		0.063
Absolute neutrophil count (/µL)	7892.16 ± 4883.07	9438.48 ± 4895.28	12903.04 ± 9494.59	9372.62 ± 6435.03		0.001
Absolute lymphocyte count (/µL)	1477.78 ± 910.88	1816.60 ± 1817.38	2056.14 ± 1883.61	1689.83 ± 1439.23		0.131
Absolute monocyte count (/µL)	876.70 ± 504.86	880.58 ± 544.57	969.30 ± 581.47	897.72 ± 529.98		0.691
MCV	88.57 ± 9.83	87.71 ± 11.40	89.18 ± 7.19	88.48 ± 9.72		0.818
MCH	28.45 ± 3.80	28.42 ± 4.44	28.78 ± 2.55	28.52 ± 3.73		0.905
MCHC	32.25 ± 1.38	32.33 ± 1.57	32.13 ± 1.29	32.65 ± 4.85		0.841
RDW	16.74 ± 3.45	16.74 ± 2.29	17.84 ± 3.52	16.97 ± 3.22		0.233
MDW	21.97 ± 5.45	28.28 ± 9.20	28.02 ± 9.01	24.90 ± 7.99		0.001

Area Under the Receiver Operator characteristic Curve (ROC-AUC) was calculated for MDW, ANC, and TLC, with p values of 0.000, 0.007, 0.005, respectively (Table 3).

**Table 3:** Area under curve; the test result variable(s): TLC and MDW have at least one tie between the positive actual state group and the negative actual state group (a). Under the nonparametric assumption; (b). Null hypothesis: true area = 0.5

Test Result Variable(s	Area	Standard Error	P-Value	95% Confidence Interval	
				Lower Bound	Upper Bound
Total Leucocyte Count (TLC)	0.633	0.046	0.005	0.543	0.723
Absolute Neutrophil Count (ANC)	0.628	0.047	0.007	0.537	0.72
Monocyte Distribution Width (MDW)	0.745	0.04	0	0.667	0.824

**Figure 1:**
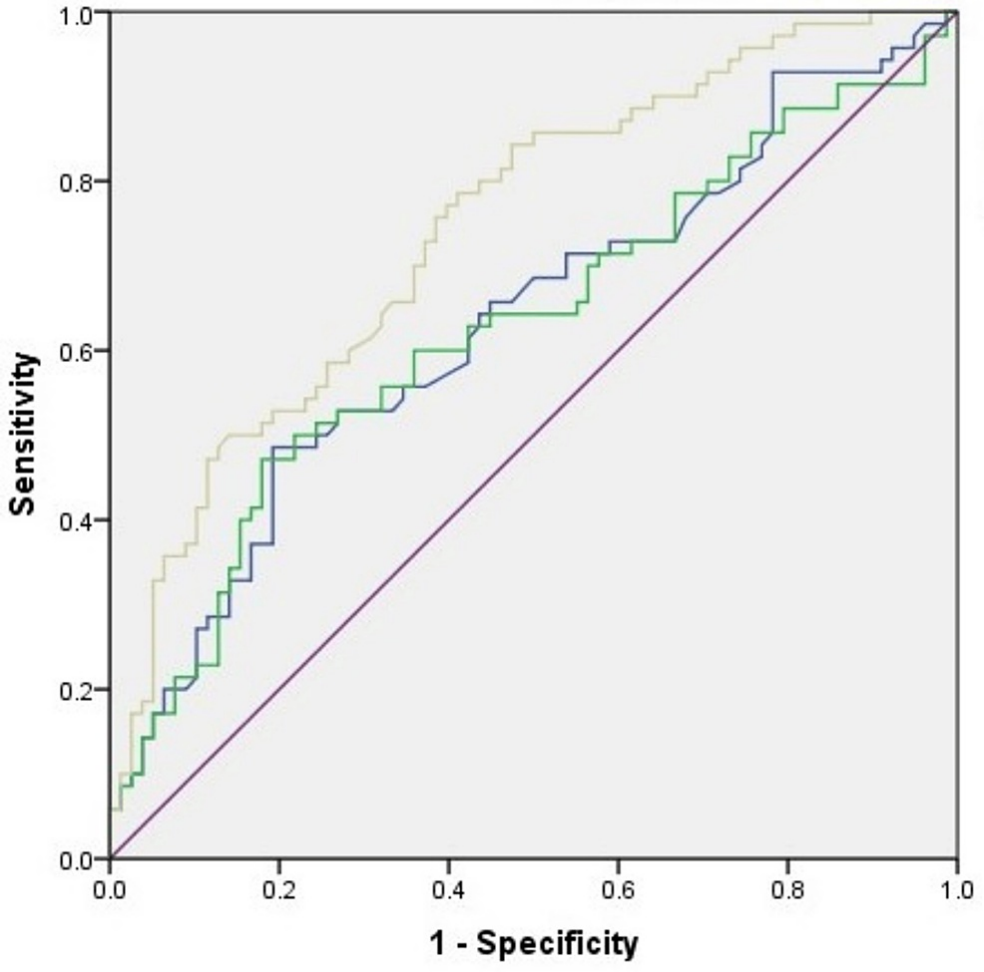
Receiver operating characteristic curve for comparison between monocyte distribution width (MDW) - Yellow; total leucocyte count (TLC) - Blue; absolute neutrophil count (ANC) - Green; and reference line - Purple

On receiver operating characteristic (ROC) curves, 0.7 is the threshold between poor and fair diagnostic accuracy. ROC analysis revealed MDW testing to be fairly accurate for the early detection of sepsis (ROC = 0.745, p = 0.001) (Figure 1).

## Discussion

This pilot study was conducted at the medicine emergency department of a premier tertiary-level center. Nearly 200 acutely ill adult patients presented daily to emergency medical services at this institute, and approximately 50% of them required admission for more than 24 hours. Of these, 10 patients were enrolled in this study on a weekly basis. MDW was evaluated as an early investigational tool for sepsis in patients being referred to premier tertiary-level hospitals.

MDW can be characteristically measured within a few minutes and without the need for additional samples and charges. A complete hemogram thus is promising as a marker of sepsis. MDW indicates volumetric changes in monocytes that are observed quite early during sepsis [15] and signal a broad spectrum of microbial pathogens causing sepsis, as corroborated in a study by Crouser et al. [10].

MDW values in patients with sepsis and with septic shock were significantly higher than in patients with non-sepsis, which was consistent with a study from Piva et al. including 506 patients where higher MDW values were found in patients with sepsis, with and without shock [16]. Further, patients who presented with sepsis and shock are associated with more co-morbidities as compared to patients with sepsis only.

Variations in monocyte volume are noticeable in reaction to pro-inflammatory signals from infectious organisms, which are referred to as pathogen-associated molecular patterns (PAMP) [17]. This determines the specificity of MDW to detect infection. Furthermore, monocytes are responsible for augmenting immune responses, thus activated monocytes, together with elevated MDW, help in the early detection of sepsis.

Limitations

This study included patients who were admitted to non-oxygen wards and hence did not require oxygenation therapy. As a result, patients with severe acute respiratory infections were not part of this study. Moreover, MDW was measured only once at admission and serial values could not be done due to logistic issues. As the study was in the emergency department, earlier established markers like C-reactive protein (CRP), erythrocyte sedimentation rate (ESR), and procalcitonin could not be done due to the short stay and unavailability of these tests at odd hours.

## Conclusions

MDW is an inexpensive and readily available biomarker that will help emergency clinicians to promptly identify sepsis accurately and start appropriate treatment in a timely manner. MDW as an early sepsis indicator would be a strong addition to current sepsis protocols, especially in resource-limited settings. It is surprising to see higher mortality in non-septic patients due to a higher prevalence of underlying chronic diseases in this study. Further studies are needed.
